# Anti-Inflammatory 8-Shogaol Mediates Apoptosis by Inducing Oxidative Stress and Sensitizes Radioresistance in Gastric Cancer

**DOI:** 10.3390/ijms26010173

**Published:** 2024-12-28

**Authors:** Tae Woo Kim, Hee Gu Lee

**Affiliations:** 1 Department of Biopharmaceutical Engineering, Dongguk University-WISE, Gyeongju 38066, Republic of Korea; tae1410@naver.com; 2 Immunotherapy Research Center, Korea Research Institute of Bioscience and Biotechnology, Yuseong-gu, Daejeon 34141, Republic of Korea

**Keywords:** 8-shogaol, PERK-CHOP pathway, apoptosis, radioresistant gastric cancer, NOX4

## Abstract

Radiotherapy is a powerful tumor therapeutic strategy for gastric cancer patients. However, radioresistance is a major obstacle to kill cancer cells. Ginger (*Zingiber officinale* Roscoe) exerts a potential function in various cancers and is a noble combined therapy to overcome radioresistance in gastric cancer radiotherapy. In this study, we suggested that 8-shogaol, a monomethoxybenzene compound extracted from *Zingiber officinale* Roscoe, has an anti-cancer and anti-inflammatory activity. In lipopolysaccharide (LPS)-induced inflammatory murine models in vivo and in vitro, 8-shogaol suppressed LPS-mediated cytokine production, including COX-2, TNFα, IL-6, and IL-1β. In xenograft mouse models of AGS gastric cancer cell lines, 8-shogaol reduced tumor volume. In gastric cancer cell lines AGS and NCI-N87, 8-shogaol reduced cell viability and increased caspase-3 activity and cytotoxicity LDH. However, combined with Z-VAD-FMK, 8-shogaol blocked caspase-dependent apoptotic cell death. 8-Shogaol induced intracellular reactive oxygen species (ROS) production, intracellular calcium (Ca^2+^) release, and endoplasmic reticulum (ER) stress response via the PERK-CHOP signaling pathway. Thapsigargin (TG), an ER stressor, mediated synergistic apoptosis and cell death in 8-shogaol-treated AGS and NCI-N87 cell lines. Nevertheless, loss of PERK or CHOP function suppressed ER-stress-induced apoptosis and cell death in 8-shogaol-treated AGS and NCI-N87 cell lines. 8-Shogaol-induced NADPH oxidase 4 (NOX4) activation is related to ROS generation. However, NOX4 knockdown and ROS inhibitors DPI or NAC blocked ER-stress-induced apoptosis by suppressing the inhibition of cell viability and the enhance of caspase-3 activity, intracellular ROS activity, and cytotoxicity LDH in 8-shogaol-treated AGS and NCI-N87 cell lines. Radioresistant gastric cancer models (AGSR and NCI-N87R) were developed and combined with 8-shogaol and radiation (2 Gy) to overcome radioresistance via the upregulation of N-cadherin and vimentin and the downregulation of E-cadherin. Therefore, these results indicated that 8-shogaol is a novel combined therapeutic strategy in gastric cancer radiotherapy.

## 1. Introduction

Globally, gastric cancer has been continuously diagnosed and reported to be a leading the high incidence rate of both women and men [1]. The tumor therapeutic direction for gastric cancer includes chemotherapy, immunotherapy, surgery, and radiotherapy [2]. Radiation therapy (radiotherapy) is the most potential tumor therapy to kill gastric cancer using particles and high-energy waves [3].

Radiotherapy exposes high-energy radiation from various sources like gamma rays, protons, and X-rays to kill cancer cells [4]. In the tumor microenvironment (TME), radioresistance is acquired by diverse factors such as the gene alterations, DNA repair system, cell cycle arrest, and autophagy [5]. In the TME, altered epithelial–mesenchymal transition (EMT) markers confer to tumor radioresistance [6]. Natural radiosensitizers such as curcumin, resveratrol, and paclitaxel are effective for sensitizing radioresistance and inducing the synergistic effect of radiotherapy [7].

8-Shogaol is a monomethoxybenzene and natural compound extracted from ginger (*Zingiber officinale* Roscoe) [8]. Gingerol’s bioactive molecules are gingerols such as 6-gingerol, 8-gingerol, and 10-gingerol and shogaol such as 6-shogaol 8-shogaol, and 10-shogaol and the dehydrated forms of shogaol have various bioactive properties, including anti-viral, anti-bacterial, anti-inflammatory, anti-tumor, and anti-allergenic efficacy [9]. Recent reports have indicated that 8-shogaol is a monomethoxybenzene of phenols and that 8-shogaol’s anti-inflammatory efficacy is mediated by the inhibition of p-nuclear factor kappa-light-chain-enhancer of activated B cells (NF-κB) and inflammatory cytokines, such as nitric oxide synthase (iNOS), cyclooxygenase-2 (COX-2), tumor necrosis factor α (TNFα), interleukin-6 (IL-6), interleukin-1β (IL-1β), and nitric oxide (NO) [10]. 8-Shogaol has also been shown to induce caspase-dependent apoptosis in human leukemia cells by producing ROS and releasing mitochondrial cytochrome c [11].

The ER is the key Ca^2+^ storage organelle, and the failure of protein folding capacity and the cytosolic release of Ca^2+^ mediates ER stress responses, inducing to the activation of the unfolded protein response (UPR) pathway [12]. The excessive or prolonged ER stress signaling pathways then regulate three UPR transmembrane sensors: activating transcription factor 6 (ATF6), PKR-like ER kinase (PERK), and inositol requiring enzyme 1α (IRE1α) [13]. The ER chaperone protein GRP78 (BIP) displaces and disrupts these proteins to modulate the interplay between unfolded proteins and the three UPR sensor proteins [14]. The activation of ER stress induces the pro-cell survival signaling pathway, but prolonged or excessive ER stress can mediate to apoptosis and cell death [15]. In addition, excessive ROS accumulation mediates oxidative stress and ER stress by producing ROS and Ca^2+^, leading to apoptosis and cell death [16]. NADPH oxidases (Noxs), particularly NOX4, confer significantly to ROS production and regulated ER stress signaling pathways [17].

In this study, we demonstrated 8-shogaol’s role in modulating anti-cancer and anti-inflammatory efficacy mediated by NOX4-induced ER stress signaling pathways and apoptosis in gastric cancer. In particular, we identified 8-shogaol’s effect to overcome radioresistant gastric cancer cells to radiotherapy.

## 2. Results

### 2.1. 8-Shogaol Inhibited Inflammation in LPS-Treated Macrophages and a Sepsis Mouse Model

**Figure 1:**
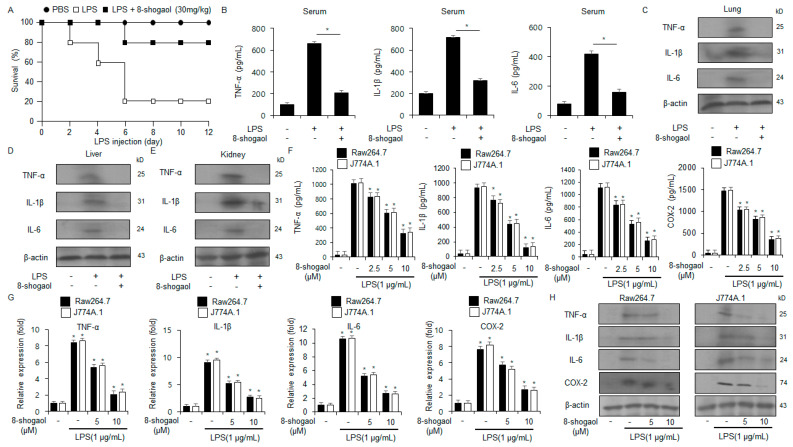
The impact of 8-shogaol on the mRNA and protein levels of inflammatory cytokines in LPS-treated macrophages. (**A**) C57BL/6 mice were administered LPS (20 mg/kg) via i.p. injection. The treatment group received 8-shogaol (30 mg/kg) via i.p. injection. The survival rate of all groups (n = 10) was analyzed daily for 12 days following LPS injection. (**B**–**E**) The protein levels of IL-1β, IL-6, and TNF-α in the serum, lung, liver, and kidney of the treated mice, as assessed by ELISA and Western blot. (**F**–**H**) The mRNA and protein levels of IL-1β, IL-6, and TNF-α in LPS (1 µg/mL)-treated Raw264.7 and J774.1 cells in the presence or absence of 8-shogaol (0, 2.5, 5, and 10 µM; 24 h), as assessed by ELISA, Western blot, and qRT-PCR. β-Actin was used to normalize the relative mRNA and protein levels. *, *p* < 0.05. All experiments were conducted three times.

To determine the anti-inflammatory effects of 8-shogaol, the LPS-induced sepsis mouse model was developed. Compared to the LPS group, the LPS + 8-shogaol group showed approximately a fourfold enhancement in survival rate (Figure 1A). We next demonstrated the protein levels of inflammatory cytokines, including IL-6, IL-1β, and TNF-α, via ELISA and Western blot. 8-Shogaol treatment notably reduced the levels of TNF-α, IL-6, and IL-1β in the kidney, lung, liver, and serum of the treated mice (Figure 1B–E). Furthermore, LPS-treated macrophages (Raw264.7 and J774.1) were used to confirm the anti-inflammatory effects of 8-shogaol. As identified by qRT-PCR, ELISA, and Western blot, 8-shogaol treatment dramatically downregulated the protein levels of inflammatory cytokines, including COX-2, IL-1β, IL-6, and TNF-α, in a dose-dependent manner (Figure 1F–H). This showed that 8-shogaol treatment effectively prevents the LPS-induced production of inflammatory cytokines in macrophages.

### 2.2. 8-Shogaol Induced Caspase-Dependent Apoptosis in Gastric Cancer Cells

**Figure 2:**
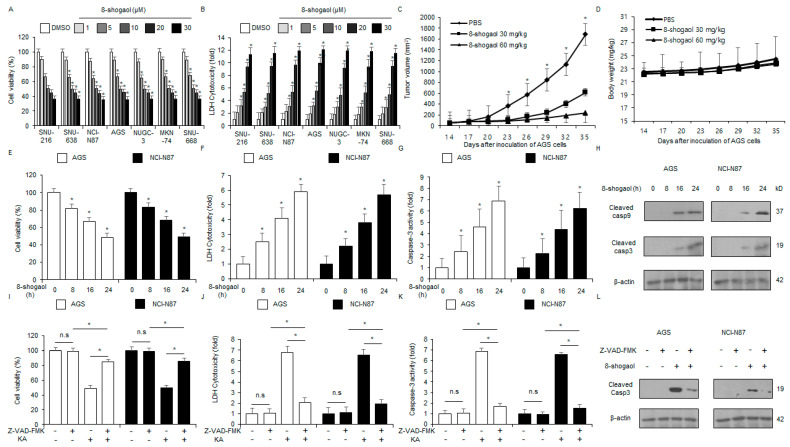
The in vitro and in vivo anti-cancer effects of 8-shogaol. (**A**,**B**) LDH and WST-1 assays were conducted for cells (SNU-216, SNU-638, NCI-N87, AGS, NUGC-3, MKN-74, and SNU-668) treated with varying doses of 8-shogaol (0, 1, 5, 10, 20, and 30 µM; 24 h). (**C**,**D**) The AGS tumor model was established by injecting 1 × 10^7^ cells into the right dorsal flank of nude mice (n = 10 per group). 8-Shogaol (30 and 60 mg/kg) was administered (i.p. injection) twice weekly; *, *p* < 0.05. The body weights of the treated mice were measured twice weekly. (**E**–**H**) NCI-N87 and AGS cells were treated with 8-shogaol for varying durations (0, 8, 16, and 24 h; 10 µM) and subjected to caspase-3, LDH cytotoxicity, and WST-1 assays. Western blot analysis of cleaved caspase-9 and -3 was also conducted following 8-shogaol treatment for the indicated durations; *, *p* < 0.05. β-actin was used as the loading control. (**I**–**L**) NCI-N87 and AGS cells were pre-treated with Z-VAD-FMK (50 μM) for 4 h before 8-shogaol treatment (10 µM, 24 h). Caspase-3 activity, LDH cytotoxicity, and WST-1 assays were performed; *, *p* < 0.05; n.s, no significance. Western blot analysis was carried out to identify the level of cleaved caspase-3. β-actin was used as the loading control.

The in vitro anti-cancer efficacy of 8-shogaol were investigated against gastric cancer cells (SNU-216, SNU-638, NCI-N87, AGS, NUGC-3, MKN-74, and SNU-668) at varying doses of 8-shogaol (1, 5, 10, 20, and 30 µM) (Figure 2A,B). The in vivo anti-tumor effects of 8-shogaol were evaluated using the AGS tumor mouse model. 8-Shogaol treatment (30 mg/kg and 60 mg/kg) significantly inhibited tumor growth compared to the control group (Figure 2C) without causing body weight loss (Figure 2D). The in vitro anti-tumor efficacy of 8-shogaol was assessed in a time-dependent manner (0, 8, 16, and 24 h; 10 µM). AGS and NCI-N87 cells were treated with 8-shogaol and subjected to LDH cytotoxicity, WST-1, and colorimetric caspase-3 activity assays. 8-Shogaol treatment indicated increased colorimetric caspase-3 activity and cytotoxicity in a time-dependent manner (Figure 2E–G). Western blot analysis also showed that 8-shogaol mediates time-dependent cleavage of caspase-3 and -9 (Figure 2H). To investigate whether 8-shogaol treatment induces apoptosis through the caspase-dependent pathway, NCI-N87 and AGS cells were co-treated with Z-VAD-FMK, a cell-permeant pan-caspase inhibitor. The 50 μM Z-VAD-FMK treatment alone did not significantly affect cytotoxicity, caspase-3 activity, or cell viability. At 10 µM 8-shogaol, however, cytotoxicity and caspase-3 activity increased. When 50 μM Z-VAD-FMK was co-treated with 10 µM 8-shogaol, cytotoxicity and colorimetric caspase-3 activity decreased significantly, leading to an enhancement in cell viability (Figure 2I–K). Western blot analysis indicated that this co-treatment reduced the level of cleaved caspase-3 compared to 8-shogaol treatment alone (Figure 2L). These results indicated that 8-shogaol suppresses the proliferation of gastric cancer cells via the caspase-related signaling pathway.

### 2.3. 8-Shogaol-Induced ER Stress Mediated Apoptosis in Gastric Cancer Cells

**Figure 3:**
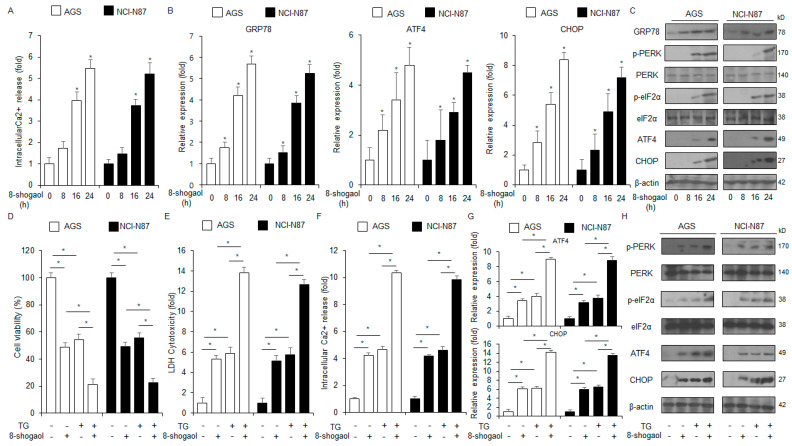
8-Shogaol induced the generation of cytosolic Ca^2+^ and apoptosis. (**A**) NCI-N87 and AGS cells were treated with 8-shogaol for varying durations (0, 8, 16, and 24 h; 10 µM) and were subjected to Ca^2+^ assay; *, *p* < 0.05. (**B**) The mRNA levels of CHOP, ATF4, and GRP78 were analyzed by qRT-PCR. β-Actin was used as the loading control. (**C**) NCI-N87 and AGS cells were treated with 8-shogaol for varying durations (0, 8, 16, and 24 h; 100 µM). Western blot analysis was carried out for the proteins associated with the ER stress signaling pathway: CHOP, ATF4, GRP78, p-eIF2α, and p-PERK. β-Actin was used as the loading control. (**D**–**F**) NCI-N87 and AGS cells were treated with 3 μM TG and 10 µM 8-shogaol for 24 h. LDH cytotoxicity, intracellular Ca^2+^, and cell viability assays were carried out; *, *p* < 0.05. (**G**,**H**) The mRNA levels of CHOP and ATF4 were analyzed by qRT-PCR. Western blot analysis was carried out to identify the protein levels of p-eIF2α, p-PERK, CHOP, and ATF4 in NCI-N87 and AGS cells treated with 10 µM 8-shogaol and 3 µM TG for 24 h. β-Actin was used as the loading control.

Ca^2+^ regulates immune evasion, cell proliferation, cell migration, survival, cell cycle arrest, and death [18]. Excessive Ca^2+^ efflux from the ER lumen into the cytosol induces ER stress, leading to apoptosis [19]. Ca^2+^ assay showed that 8-shogaol treatment increased Ca^2+^ production in NCI-N87 and AGS cells in a time-dependent manner (Figure 3A). Moreover, qRT-PCR analysis of these cells indicated that 8-shogaol treatment enhanced the mRNA levels of CHOP, GRP78, and ATF4 in a time-dependent manner; Western blot analysis indicated that the protein levels of p-eIF2α, p-PERK, CHOP, ATF4, and GRP78 increased with 8-shogaol treatment (Figure 3B,C). To investigate whether ER stress is associated with 8-shogaol-mediated apoptosis, the cells were co-treated with 8-shogaol and the ER stressor TG. This co-treatment synergistically enhanced the intracellular Ca^2+^ release and cytotoxicity (Figure 3D–F). In addition, the levels of CHOP, ATF4, p-eIF2α, and p-PERK also significantly enhanced following the treatment (Figure 3G,H).

### 2.4. PERK or CHOP Silencing Inhibited 8-Shogaol-Induced Apoptosis in Gastric Cancer Cells

**Figure 4:**
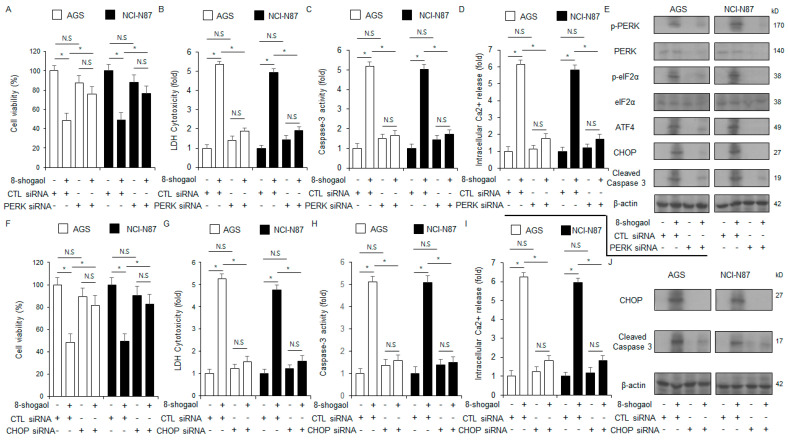
PERK silencing suppressed 8-shogaol-mediated apoptosis in gastric cancer cells. (**A**–**E**) PERK siRNA was transfected into NCI-N87 and AGS cells. The cells were then treated with 10 µM 8-shogaol for 24 h. Cytosolic Ca^2+^, caspase-3 activity, LDH cytotoxicity, and WST-1 assays were carried out; *, *p* < 0.05; N.S, no significance. Western blot analysis was carried out to identify the protein levels of cleaved caspase-3, CHOP, ATF4, p-eIF2α, and p-PERK in NCI-N87 and AGS cells treated with 10 µM 8-shogaol for 24 h. β-Actin was used as the loading control. (**F**–**J**) CHOP siRNA was transfected into NCI-N87 and AGS cells. The cells were then treated with 10 µM 8-shogaol for 24 h. Cytosolic Ca^2+^, caspase-3 activity, LDH cytotoxicity, and WST-1 assays were carried out; *, *p* < 0.05; N.S, no significance. Western blot analysis was carried out to identify the protein levels of cleaved caspase-3, as well as CHOP, in NCI-N87 and AGS cells treated with 10 µM 8-shogaol for 24 h. β-Actin was used as the loading control.

To assess whether 8-shogaol-induced apoptosis is dependent on PERK or CHOP, NCI-N87 and AGS cells were transfected with siRNAs for the corresponding genes. PERK silencing reduced 8-shogaol-induced increase in caspase-3 activity, intracellular Ca^2+^ activity, and cytotoxicity (Figure 4A–D). Western blot analysis showed that p-PERK, p-eIF2α, cleaved caspase-3, ATF4, and CHOP levels were also reduced after PERK silencing in 8-shogaol-treated NCI-N87 and AGS cells (Figure 4E). CHOP silencing also decreased 8-shogaol-mediated increases in caspase-3 activity, intracellular Ca^2+^ activity, and cytotoxicity (Figure 4F–I). Western blot analysis showed that CHOP knockdown decreased the levels of cleaved caspase-3, as well as CHOP, in 8-shogaol-treated NCI-N87 and AGS cells (Figure 4J). These findings identified that 8-shogaol-mediated apoptosis in gastric cancer cells is mediated by ER stress.

### 2.5. Nox4 Silencing Inhibited 8-Shogaol-Induced Apoptosis in Gastric Cancer Cells

**Figure 5:**
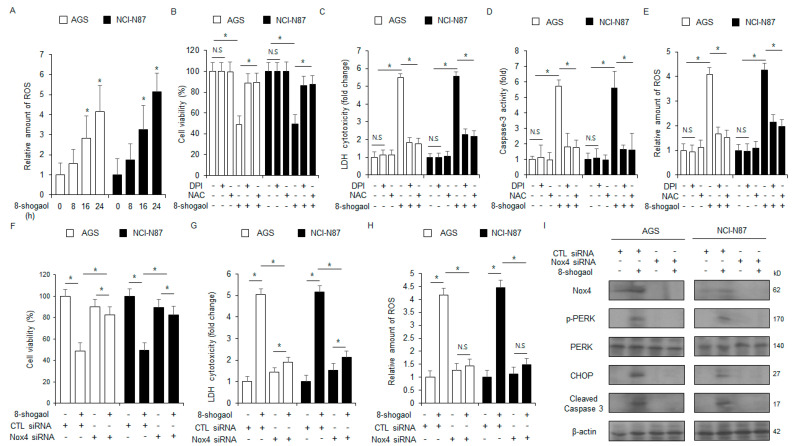
NOX4 silencing suppresses ROS-mediated ER stress and apoptosis in 8-shogaol-treated gastric cancer. (**A**) NCI-N87 and AGS cells were treated with 10 µM 8-shogaol for the indicated durations and subjected to intracellular ROS assay DCFDA; *, *p* < 0.05. (**B**–**E**) NCI-N87 and AGS cells were treated with 10 µM 8-shogaol, 1 µM DPI, and 100 µM NAC for 24 h. Caspase-3 activity, LDH cytotoxicity, intracellular ROS, and WST-1 assays were carried out; *, *p* < 0.05; N.S, no significance. (**F**–**I**) NOX4 siRNA was transfected into NCI-N87 and AGS cells and then treated with 10 µM 8-shogaol for 24 h. Cytosolic ROS, WST-1, and LDH cytotoxicity assays were carried out; *, *p* < 0.05; N.S, no significance. Western blot analysis was carried out to identify the protein levels of cleaved caspase-3, CHOP, NOX4, PERK, and p-PERK in NCI-N87 and AGS cells treated with 10 µM 8-shogaol for 24 h. β-Actin was used as the loading control.

We next investigated the impact of 8-shogaol treatment on intracellular ROS release. 8-Shogaol treatment increased the level of ROS production in gastric cancer cells over varying treatment times (Figure 5A). Next, NCI-N87 and AGS cells were treated with ROS inhibitors (DPI or NAC) in the presence of 8-shogaol. This co-treatment with 8-shogaol-mediated enhancements in cytotoxicity, intracellular ROS generation, and caspase-3 activity (Figure 5B–E). Next, the cells were transfected with a NOX4-specific siRNA and treated with 8-shogaol. NOX4 silencing decreased the enhancements in intracellular ROS and cytotoxicity induced by 8-shogaol treatment (Figure 5F–H). Western blot analysis showed that NOX4 silencing also suppressed the enhancements in the levels of CHOP, cleaved caspase-3, p-PERK, and NOX4 (Figure 5I). These results showed that NOX4 confers to 8-shogaol-mediated intracellular ROS release and apoptosis in gastric cancer cells.

### 2.6. 8-Shogaol Sensitized Radio-Resistant Gastric Cancer Cells to Radiotherapy by Modulating EMT Markers

**Figure 6:**
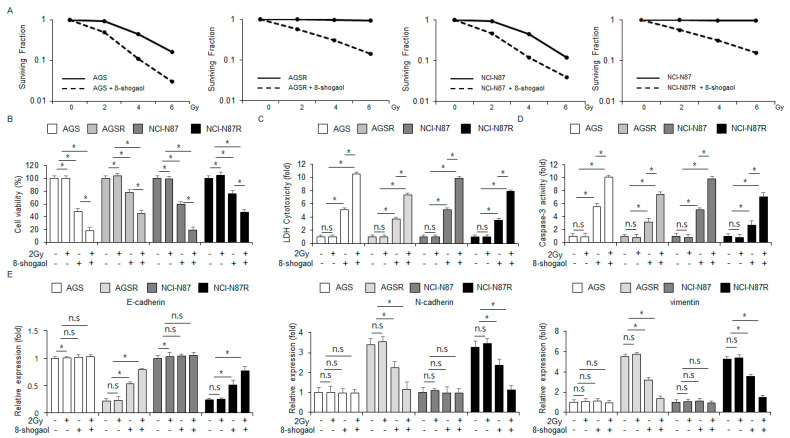
Radiation combined with 8-shogaol overcame radioresistance in gastric cancer cells. (**A**) Colony formation analysis was carried out for NCI-N87, AGS, NCI-N87R, and AGSR cells following radiation at varying intensities (0, 2, 4, and 6 Gy) and/or 8-shogaol treatment. The cell survival rate was quantified. (**B**–**D**) NCI-N87, AGS, NCI-N87R, and AGSR cells treated with 10 µM 8-shogaol and 2 Gy radiation for 24 h were subjected to caspase-3 activity, WST-1, and LDH cytotoxicity assays; *, *p* < 0.05; n.s, no significance. (**E**) The mRNA levels of vimentin, N-cadherin, and E-cadherin were analyzed by qRT-PCR in NCI-N87, AGS, NCI-N87R, and AGSR cells treated with 10 µM 8-shogaol and 2 Gy radiation for 24 h; *, *p* < 0.05; n.s, no significance. β-actin was used as the loading control.

Although radiotherapy is a potential tumor therapeutic strategy to sensitize gastric cancer cells, it frequently causes radioresistance [20]. Next, we assessed whether 8-shogaol overcomes radio-resistant NCI-N87R and AGSR cells using a clonogenic cell assay. 8-Shogaol treatment was synergized with varying radiation intensities (2, 4, and 6 Gy) to enhance cytotoxic effects against NCI-N87R, AGSR, NCI-N87, and AGS cells (Figure 6A). In NCI-N87 and AGS cells, 8-shogaol enhanced caspase-3 activity and cytotoxicity. The 2 Gy radiation combined with 8-shogaol further enhanced these properties and significantly decreased cell viability, with radiation alone having no significant effects (Figure 6B–D). In NCI-N87R and AGSR cells, 8-shogaol treatment again increased cytotoxicity and caspase-3 activity, leading to synergistic anti-cancer effects when combined with 2 Gy radiation; radiation alone had no significant effects in these cells (Figure 6B–D). To investigate whether this combination regulates the EMT phenomenon, the radio-resistant gastric cancer cells were subjected to qRT-PCR analysis. Both 8-shogaol alone and co-treatment with 2 Gy radiation increased the mRNA level of E-cadherin and decreased the mRNA levels of vimentin and N-cadherin in NCI-N87R and AGSR cells. On the other hand, the non-resistant cells showed no significant changes in these mRNA levels (Figure 6E). These results suggested that combining 8-shogaol with radiation may be an effective strategy for treating radio-resistant gastric cancer.

## 3. Discussion

Recent studies have reported that natural compounds, including phenols, flavonoids, terpenoids, and alkaloids, display powerful anti-tumor efficacy [21,22]. In various cancer cell types, these compounds often mediate intracellular ROS and Ca^2+^ release, thereby causing oxidative-stress-driven apoptosis [23,24]. In this study, we explored 8-shogaol’s anti-tumor effects and ability to sensitize radio-resistant breast cancer cells to radiotherapy. Our findings identified that 8-shogaol’s anti-tumor efficacy induce apoptosis in gastric cancer cells both in vitro and in vivo, highlighting its clinical potential to inhibit tumor growth [25]. By co-treating Z-VAD-FMK and 8-shogaol, we identified that 8-shogaol-mediated apoptosis in gastric cancer cells is dependent on caspase activity. Moreover, 8-shogaol increased ER stress in these cells, thereby activating the downstream ER stress sensors, IRE1α, PERK, and ATF6, inducing apoptotic cell death [26]. Upon UPR induction, the ER chaperone protein GRP78 dissociated from these sensors mediated the phosphorylation of eIF2α and PERK [27]. It has been identified that p-eIF2α mediates the activation of cytosolic ATF4. The translocated nuclear ATF4 binds to the promoter of nuclear CHOP and induces its expression [28]. 8-Shogaol was found to induce the ER stress signaling pathway by promoting the generation of ROS and cytosolic Ca^2+^. This process ultimately mediated apoptosis through the PERK-ATF4-CHOP signaling pathway. Silencing PERK or CHOP in these cells led to the suppression of 8-shogaol-caused apoptosis. When 8-shogaol was co-treated with TG, synergistic apoptosis was mediated via the PERK-ATF4-CHOP axis in gastric cancer cells. In this phenomenon, NOX4 was demonstrated as a regulator of ROS generation promoted by 8-shogaol treatment. The activation of NOX4 was found to mediate ER-stress-induced apoptosis by promoting ROS and intracellular Ca^2+^ release in 8-shogaol-treated cells. NOX4 silencing or ROS inhibitor (NAC or DPI) treatment inhibited the increase in ROS, caspase-3 activity, cytotoxicity, and the PERK-ATF4-CHOP signaling pathway in 8-shogaol-treated gastric cancer cells. Although radiotherapy is a primary cancer treatment option, tumor cells often develop radioresistance after exposure, reducing the effectiveness of therapy [29]. Recent reports have indicated that natural compounds, including withaferin A, celastrol, ursolic acid, zerumbone, C-phycocyanin, emodin, flacopiridol, and berberine, are effective radiotherapy sensitizers with minimal side effects [30,31]. The EMT regulatory proteins have been related to the development of radioresistance, chemoresistance, and hypoxic environments [32]. The development of anti-cancer agents that can sensitize radio-resistant cancer cells has the possibility to increase the effectiveness of radiotherapy. When radiation was combined with 8-shogaol, the radio-resistant NCI-N87R and AGSR cells effectively overcame radiotherapy. This phenomenon was indeed identified to be dependent on the change of EMT-regulatory proteins, such as N-cadherin, vimentin, and E-cadherin.

Various natural compounds have powerful anti-tumor effects in many cancer types [33]. Kaempferol has been shown to mediate apoptosis by promoting the cleavage of caspase-3 and G_2_/M phase cell cycle arrest in human gastric cells [34]. The monomeric compound alternol has been found to promote apoptosis and ROS-dependent ER stress via the PERK-ATF4-CHOP axis and the IRE1α–XBP1-CHOP axis in prostate cancer cells, PC-3, 22RV1, and C4-2, where the NF-κB inhibitor Imoxin and SN50 treatment were able to suppress alternol-mediated ER stress and apoptosis [35]. Moreover, catechol has been identified to display anti-tumor efficacy and the ability to sensitize radio-resistant cells through the change of EMT markers such as vimentin, Snail, and E-cadherin in pancreatic cancer cells [36]. Analogously to these natural compounds, we identified that 8-shogaol induces apoptosis in gastric cancer cells through the PERK-ATF4-CHOP axis. Importantly, 8-shogaol was demonstrated to powerfully sensitize radio-resistant cells by regulating the aforementioned EMT-related proteins, including vimentin, N-cadherin, and E-cadherin. Recently, it has been demonstrated that 8-shogaol induces caspase-3-dependent apoptosis via the activation of ROS-mediated ER stress in human oral cells [37].

ROS release and mitochondrial dysfunction induce apoptosis by activating the PERK-ATF4-CHOP axis in many cancers [38]. Gossypol has been shown to induce apoptosis in human pancreatic cancer cells BxPC-3 and MIA PaCa-2 cells through the CHOP-DR5 axis [39]. NOXs are potential enzymes that produce ROS and serve as important characteristics of cell metabolism in cancer [40]. The seven transmembrane NOX family, including dual oxidases 1 and 2 and NOX1-5, generate superoxide anion radicals and play potential roles in various cancers [41]. NOX4-mediated ROS generation induces apoptotic cell death and plays a potential role in regulating cell survival and death [42]. Cannabidiol has been found to induce NOX4-mediated release of mitochondrial ROS and promote caspase-dependent apoptosis through the activation of ER stress signaling pathway and the inhibition of the mTOR signaling pathway in breast cancer cells [43]. Similarly, we identified that 8-shogaol mediates apoptosis via the PERK-ATF4-CHOP axis driven by NOX4-caused release of ROS. Silencing NOX4 or ROS inhibitors (DPI or NAC) treatment inhibited the increases in ROS, cytotoxicity, and caspase-3 activity in 8-shogaol-treated gastric cancer cells, meaning NOX4 as the regulator of 8-shogaol-mediated ROS production.

Radiotherapy utilizes high-energy beams to kill cancer cells, but gastric cancer patients often acquire radioresistance [44]. Therefore, strategies to overcome resistant cancer cells to radiation are significantly needed [45]. Radiation treatment decreases oxygen levels in the TME, leading to a hypoxic condition that mediates radioresistance by modulating EMT [46]. In this process, the epithelial cell regulatory protein E-cadherin is downregulated, while mesenchymal cell regulatory proteins such as vimentin and N-cadherin are upregulated [47]. In radio-resistant NCI-N87R and AGSR cells, 8-shogaol combined with radiation blocked these phenomena, suppressing radioresistance. Ultimately, 8-shogaol combined with radiation significantly increased ER stress and apoptosis by modulating EMT-related proteins in radio-resistant gastric cancer cells

## 4. Materials and Methods

### 4.1. Reagents

8-Shogaol (PHL83910), N-acetylcysteine (NAC), diphenyleneiodonium (DPI), lipopolysaccharide (LPS; L4391), Z-VAD-FMK, and TG (T9033) were purchased from Sigma-Aldrich (St. Louis, MO, USA).

### 4.2. Cell Culture

Human gastric cancer cell line (SNU-216, SNU-638, NCI-N87, AGS, NUGC-3, MKN-74, and SNU-668) was purchased from the American Type Culture Collection (ATCC, Manassas, VA, USA) and the Korean Cell Line Bank (Seoul, Republic of Korea) and then cultured at 37 °C under 5% CO_2_ in Dulbecco’s modified Eagle’s medium (DMEM; Welgene, Gyeongsan-si, Gyeongsangbuk-do, Republic of Korea) supplemented with 10% fetal bovine serum (FBS; Welgene) and 1% penicillin–streptomycin (PS; Welgene).

### 4.3. Cell Viability and Proliferation Assays

To identify the effect of 8-shogaol on the viability of treated cells, the WST-1 assay (Roche Applied Science, Indianapolis, IN, USA) was conducted in both treatment time- and concentration-dependent manners (8-shogaol; 1, 5, 10, 20, and 30 µM; 24 h). Gastric cancer cells were seeded and cultured in a 96-well cell culture plate (1 × 10^4^ cells/well). Following the manufacturer’s protocol, the absorbance at 450 nm was measured from each well using a microplate reader (Molecular Devices, San Jose, CA, USA).

### 4.4. Lactate Dehydrogenase (LDH) Cytotoxicity Assay

To determine the cytotoxicity of 8-shogaol against breast cancer cells, LDH cytotoxicity assay (Abcam, Cambridge, MA, USA) was conducted in both treatment time- and concentration-dependent manners. Gastric cancer cells were seeded and cultured in a 96-well cell culture plate (1 × 10^4^ cells/well). Following the manufacturer’s protocol, the absorbance at 490 nm was measured from each well using a microplate reader.

### 4.5. Colorimetric Caspase-3 Activity Assay

To identify 8-shogaol’s impact on caspase-3 activity in gastric cancer cells, a colorimetric caspase-3 activity assay (Abcam) was conducted in both treatment time- and concentration-dependent manners. Gastric cancer cells were seeded and cultured in a 96-well cell culture plate (1 × 10^4^ cells/well). Following the manufacturer’s protocol, the absorbance at 490 nm was measured from each well using a microplate reader.

### 4.6. Intracellular Ca^2+^ Assay

To identify the level of the intracellular Ca^2+^ in gastric cancer cells treated with 8-shogaol, an intracellular Ca^2+^ assay (Abcam) was carried out in both treatment time- and concentration-dependent manners. Gastric cancer cells were seeded and cultured in a 96-well cell culture plate (1 × 10^4^ cells/well). Following the manufacturer’s protocol, the fluorescence at 575 nm was measured from each well using FilterMax F5 (Molecular Devices).

### 4.7. Intracellular ROS Assay

To identify the level of ROS in gastric cancer cells treated with 8-shogaol, an intracellular ROS assay (Abcam) was carried out in both treatment time- and concentration-dependent manners. Gastric cancer cells were seeded and cultured in a 96-well cell culture plate (1 × 10^4^ cells/well). Following the manufacturer’s protocol, the fluorescence at 605 nm (excitation at 520 nm) was measured from each well using FilterMax F5.

### 4.8. Radio-Resistant NCI-N87R and AGSR Cell Lines

NCI-N87 and AGS cells were cultured in 60 mm culture plates. The cells were irradiated at 4 Gy daily for 90 days to establish radio-resistant NCI-N87R and AGSR cell lines.

### 4.9. Radiotherapy

NCI-N87, AGS, NCI-N87R, and AGSR cells were cultured in 60 mm culture plates at 37 °C under 5% CO_2_. The cells were irradiated using an irradiator with a cesium-137 source (Atomic Energy of Canada, Ltd., Mississauga, ON, Canada).

### 4.10. Clonogenic Cell Assays

NCI-N87, AGS, NCI-N87R, and AGSR cells were cultured in 60 mm culture plates at 37 °C under 5% CO_2_ for colony formation. The colonies were stained with 0.5% crystal violet solution (Sigma-Aldrich, St. Louis, MO, USA).

### 4.11. Transfection Assay

Small interfering RNAs (siRNAs) for CHOP (Bioneer, Daejeon, Republic of Korea), Nox4 (Santacruz, Dallas, TX, USA), and PERK (Santacruz) were used for gene-silencing experiments. NCI-N87 and AGS cells were first seeded in 6-well culture plates. Following the manufacturer’s protocol, the cells were transfected with siRNAs (30 nmol/mL). Lipofectamine 2000 (Invitrogen, Carlsbad, CA, USA) was used as the transfection agent for siRNAs.

### 4.12. Protein and RNA Purification

Gastric cancer cells were cultured in 100 mm culture plates at 37 °C under 5% CO_2_. Following the manufacturer’s protocol, total RNA and protein were isolated with Trizol reagent (Invitrogen) and extracted using the radio-immunoprecipitation assay (RIPA) lysis buffer (Thermo Fisher Scientific, Waltham, MA, USA).

### 4.13. Quantitative Reverse Transcription Polymerase Chain Reaction (qRT PCR) and Western Blot Analyses

For qRT-PCR assay, all reactions were performed in triplicate. The primers were adopted from a study by Kim et al. and purchased from Bioneer (Daejeon, Republic of Korea) [48]. To determine relative gene expression levels, the 2^−ΔΔCt^ method was used. To identify protein expression levels, Western blot analysis was performed. After separating the proteins via SDS-PAGE, they were transferred to PVDF membranes, which were blocked with 5% skim milk and incubated with primary antibodies. The primary antibodies used were as follows: cleaved caspase-9 (Cell Signaling, Danvers, MA, USA), CHOP (Cell Signaling), ATF4 (Cell Signaling), p-PERK (Thr980) (Cell Signaling), cleaved caspase-3 (Cell Signaling), p-eIF2ɑ (Ser51) (Cell Signaling), PERK (Cell Signaling), eIF2α (Santa Cruz, CA, USA), GRP78 (Santa Cruz), β-actin (Santa Cruz), and Nox4 (Proteintech, Rosamond, IL, USA). HRP-conjugated secondary antibodies were used with an anti-rabbit IgG HRP-linked antibody (Santa Cruz) and m-IgGK BP-HRP-linked antibody (Santa Cruz). The membranes were visualized using a chemiluminescent substrate for HRP (MilliporeSigma, Burlington, MA, USA).

### 4.14. Tumor and Inflammation Mouse Models

All mouse models were stabilized using female mice. Five-week-old mice were obtained from OrientBio, Inc. (Daejeon, Republic of Korea), and acclimated in a sterile room for one week on an NIH-7 open formula diet. The mice were then divided randomly into three groups. All mouse experiments were conducted in accordance with the guidelines of the Kyung-Hee University Animal Care and Use Committee. The AGS tumor model was established by subcutaneously injecting 1 × 10^7^ cells suspended in PBS into the right dorsal flanks of athymic BALB/c nude mice (*nu*/*nu*). When the average tumor volume reached 200 mm^3^, the mice were randomly divided into three groups (n = 10): control (PBS), 30 mg/kg 8-shogaol, and 60 mg/kg 8-shogaol. The specified doses of 8-shogaol or PBS were administered by intraperitoneal (i.p.) injection twice weekly. Tumor volume (mm^3^) was calculated using the following formula: (*L* × *W*^2^)/2, where *L* is the length and *W* is the width of the tumor (mm^3^). The anti-inflammatory effects of 8-shogaol were assessed using the LPS-induced inflammation model. The mice were randomly divided into three groups: PBS, LPS, and LPS + 8-shogaol. The groups receiving LPS were administered 20 mg/kg of LPS by i.p. injection. The LPS + 8-shogaol group was administered with 30 mg/kg of 8-shogaol by i.p. injection twice weekly. The survival rate was monitored for 12 days following LPS injection, and blood and tissues were collected for analysis.

### 4.15. Cytokine Levels

Raw264.7 and J774.1 cells were seeded in 96-well culture plates (1 × 10^4^ cells/well). The cells were treated with 1 μg/mL LPS in the absence or presence of 8-shogaol (0, 2.5, 5, and 10 μM; 24 h) for 24 h. The protein levels of IL-6, IL-1β, and TNF-α were assessed via enzyme-linked immunosorbent assay (ELISA) Following the manufacturer’s protocol: IL-6 (DY-406; R&D Systems, Minneapolis, MN, USA), IL-1β (DY-401; R&D Systems), and TNF-α (DY-410; R&D Systems).

### 4.16. Statistical Analysis

All experiments were conducted at least three times. Statistical significance was analyzed using analysis of variance (ANOVA) and Student’s *t*-test, with a *p*-value < 0.05 considered statistically significant.

## 5. Conclusions

In conclusion, we demonstrated 8-shogaol’s anti-cancer and anti-inflammatory properties both in vitro and in vivo. 8-Shogaol treatment was shown to mediate apoptosis through the PERK-ATF4-CHOP axis. Moreover, the treatment increased the production of ROS and intracellular Ca^2+^ and upregulated NOX4 in gastric cancer cells. When 8-shogaol was combined with radiation, radioresistance was effectively sensitized via the EMT change in NCI-N87R and AGSR cells.

## Data Availability

Data are contained within the article.
